# Association between the Pro12Ala Polymorphism of the Peroxisome Proliferator-Activated Receptor Gamma Gene and Strength Athlete Status

**DOI:** 10.1371/journal.pone.0067172

**Published:** 2013-06-14

**Authors:** Agnieszka Maciejewska-Karlowska, Marek Sawczuk, Pawel Cieszczyk, Aleksandra Zarebska, Stanislaw Sawczyn

**Affiliations:** 1 Department of Biological Bases of Physical Culture, Faculty of Physical Education and Health Promotion, University of Szczecin, Szczecin, Poland; 2 Department of Genetics, Faculty of Biology, University of Szczecin, Szczecin, Poland; 3 Department of Sport, Faculty of Tourism and Recreation, Academy of Physical Education and Sport, Gdansk, Poland; Morehouse School of Medicine, United States of America

## Abstract

**Background:**

The 12Ala allele of the Peroxisome Proliferator-Activated Receptor gamma gene (*PPARG*) Pro12Ala polymorphism produces a decreased binding affinity of the PPARγ2 protein, resulting in low activation of the target genes. The 12Ala allele carriers display a significantly improved insulin sensitivity that may result in better glucose utilisation in working skeletal muscles. We hypothesise that the *PPARG* 12Ala allele could be associated with strength athlete status in Polish athletes.

**Methodology:**

The genotype distribution of *PPARG* Pro12Ala was examined in 660 Polish athletes. The athletes were stratified into four subgroups: *endurance*, *strength-endurance*, *sprint-strength* and *strength*. Control samples were prepared from 684 unrelated sedentary volunteers. A χ^2^ test was used to compare the *PPARG* Pro12Ala allele and genotype frequencies between the different groups of athletes and control subjects. Bonferroni’s correction for multiple testing was applied.

**Results:**

A statistically significant higher frequency of *PPARG* 12Ala alleles was observed in the subgroup of *strength* athletes performing short-term and very intense exertion characterised by predominant anaerobic energy production (13.2% vs. 7.5% in controls; *P* = 0.0007).

**Conclusion:**

The *PPARG* 12Ala allele may be a relevant genetic factor favouring strength abilities in professional athletes, especially in terms of insulin-dependent metabolism, a shift of the energy balance towards glucose utilisation and the development of a favourable weight-to-strength ratio.

## Introduction

Physical performance phenotypes depend on combination of environmental as well as genetic factors and are characterized as quantitative and multifactorial. Interaction effects between genes and the environment and the identification of genes or coding variants in relation to athletes’ characteristics are at present the matter of investigation worldwide. Many genes have been investigated for their potential contributions to human variation in fitness, performance or trainability. One of the genetic loci shown to be related to physical performance is the Peroxisome Proliferator-Activated Receptor Gamma gene (*PPARG*) that encodes the PPARγ protein - a transcriptional regulator involved in energy control and lipid/glucose homeostasis. Although the natural compounds acting as specific PPARγ ligands are not yet firmly established, endogenous polyunsaturated fatty acids (PUFA), eicosanoids derived from nutrition or metabolic pathways and some synthetic agonists (such as thiazolidinediones) can activate PPARγ [1]. PPARγ induction enables its binding (as an obligate heterodimer with retinoic acid X receptor) to the PPRE (peroxisome proliferator response element) sequences in the promoter regions of target genes to regulate their transcription. PPARγ thus controls the expression of various genes engaged in different metabolic pathways. PPARγ is highly expressed in adipocytes, serves as a critical regulator of fat cell differentiation and promotes the formation of mature triglyceride-rich adipocytes [2]. PPARγ is also known as a modulator of insulin-signalling pathways acting through very complicated metabolic networks. This nuclear receptor may sensitise skeletal muscle and liver to the actions of insulin; however, the mechanism behind the insulin-sensitising effect has remained elusive. The most surprising finding is that PPARγ can improve insulin sensitivity via both PPARγ supraphysiological stimulation using antidiabetic drugs and the reduction of PPARγ activity resulting from *PPARG* gene knockout or polymorphism [3], [4]. Thus, PPARγ appears to be a key regulator of adipogenesis, fatty acid storage and energy balance. Due to PPARγ’s role in controlling lipid/glucose metabolism, it is regarded as a physiological factor associated with predispositions to hyperlipidemia, insulin resistance, type 2 diabetes mellitus, obesity and cardiovascular diseases (for a review, see [5]). PPARγ is also involved in the inhibition of osteoblast differentiation and osteoclastogenesis [6], [7], indicating a completely new physiological role for this nuclear receptor that is not related to lipid and glucose homeostasis.

In humans, PPARγ is encoded by the *PPARG* gene located on chromosome 3. Differential *PPARG* promoter usage and alternative splicing produces different mRNAs [8], including at least four transcripts (PPARG1, PPARG2, PPARG3 and PPARG) that differ at their 5-prime ends [8], [9], [10]. However, the protein sequences of PPARγ1, γ3 and γ4 are identical (this sequence is encoded by exons 1 to 6 of the *PPARG* gene), while the PPARγ2 protein contains 28 additional amino acids at the N-terminus that are encoded by the exon B fragment of the *PPARG* gene [2]. Studies in rats revealed that PPARγ1 transcripts are expressed in the spleen, intestine and white adipose tissue, while the PPARγ2 isoform is preferentially expressed in the adipocytes of white and brown fat [11].

The C34G substitution (rs1801282) is located within the exon B sequence of the *PPARG* gene, resulting in the Pro12Ala polymorphism described in the PPARγ2 protein [12]. The 12Ala allele shows a decreased binding affinity of the PPARγ2 protein to the PPRE sequences in responsive promoter regions, resulting in low activation of target genes [13], [14]. Medical studies on this polymorphic site revealed that it is associated with cardiovascular disorders [15] as well as type 2 diabetes [16], insulin resistance [17] and obesity [18]. The whole-body insulin sensitivity measurements revealed that 12Ala allele carriers displayed a significantly improved insulin sensitivity [19] that may result in better glucose utilisation in working skeletal muscles [20], [21].

Because physical fitness largely depends on the balance between lipid-carbohydrate metabolism and precise substrate usage, the PPAR transcriptional factors and their co-activators are an area of interest to sport scientists. We have recently demonstrated that the *PPARA* intron 7 and *PPARGC1A* Gly482Ser polymorphisms are associated with endurance athlete status in Polish athletes, indicating the relevance of PPARα and PGC-1α in athletic performance [22], [23], [24]. Taking into account the physiological role of the PPARγ protein, the *PPARG* Pro12Ala polymorphism can also be considered a genetic factor that contributes to the polygenic profile of athletic performance. Only two studies regarding the association of the *PPARG* Pro12Ala polymorphism with locomotor activity and aerobic and anaerobic performance in athletes have been published, both by Ahmetov and his colleagues [25], [26]. An analysis of the correlation between *PPARG* Pro12Ala genotypes and physical performance was conducted on a large group of Russian athletes representing different disciplines: strength-endurance athletes [25] and sprint and strength athletes [26]. The frequency of the 12Ala allele was significantly higher in the studied athlete groups compared to sedentary controls; moreover, a hypertrophic effect of the 12Ala allele on muscle fibres was observed [26].

Thus, we expected the *PPARG* 12Ala allele to be associated with strength athlete status in Polish athletes. To test this hypothesis, we decided to perform a genetic association study that aimed to detect a correlation between the selected genetic polymorphism and athletic status. Therefore, we examined the genotype distribution of the *PPARG* Pro12Ala allele in a group of Polish athletes that were divided into four subgroups (*endurance*, *strength-endurance*, *sprint-strength* and *strength* athlete subgroups), from the more endurance-oriented to the more strength-oriented, according to the following values: relative aerobic/anaerobic energy system contribution, time of competitive exercise performance, and intensity of exertion in each sport. These groups represented the whole human physical performance continuum. Our study indicate a statistically significant higher frequency of the *PPARG* 12Ala allele (compared to sedentary controls) in the subgroup of *strength* athletes performing short-term and very intense exertion characterised by predominant anaerobic energy production.

## Materials and Methods

### Ethics Statement

The procedures followed in the study were conducted ethically according to the principles of the World Medical Association Declaration of Helsinki and ethical standards in sport and exercise science research. The procedures followed in the study were approved by the Pomeranian Medical University Ethics Committee (approval nr BN-001/45/08). All participants were given a consent form and a written information sheet concerning the study, providing all pertinent information (purpose, procedures, risks, benefits of participation). The potential participant had time to read the information sheet and the consent form. After ensuring that the participant has understood the information every participant gave written informed consent (signed consent form) to genotyping on the understanding that it was anonymous and that the obtained results would be confidential. The experimental procedures were conducted in accordance with the set of guiding principles for reporting the results of genetic association studies defined by the STrengthening the REporting of Genetic Association studies (STREGA) Statement [27].

### Participants

The association study used 660 Polish athletes (age 28.8±6.5 years, male *n* = 462 and female *n* = 198). The athletes were prospectively stratified into four groups according to the values of relative aerobic/anaerobic energy system contribution, time of competitive exercise performance and intensity of exertion in each sport. The first group, designated *endurance* athletes, included athletes (*n* = 120) with predominant aerobic energy production (duration of exertion over 30 minutes, intensity of exertion moderate). This group included triathletes (*n* = 32), race walkers (*n* = 10), road cyclists (*n* = 35), 15–50 km cross-country skiers (*n* = 11) and marathon runners (*n* = 32). The second group, designated *strength-endurance* athletes (*n* = 162), comprised athletes whose sports utilise mixed anaerobic/aerobic energy production, with a duration of exertion ranging from 5 to 30 minutes and a moderate to high intensity of exertion. This group included rowers (*n* = 110), 3–10 km runners (*n* = 28) and 800–1500 m swimmers (*n* = 24). The third group (*sprint-strength* athletes; *n* = 196) also included athletes with mixed energy production, but with a shorter competitive exercise performance time than the second subgroup (1–5 minutes; for combat sports, the duration of a single bout of competition was considered), though the intensity of exertion was higher and the balance between anaerobic/aerobic energy production was shifted towards the anaerobic system. This group comprised kayakers (*n* = 17), 800–1500 m runners (*n* = 16), 200–400 m swimmers (*n* = 13), judokas (*n* = 28), wrestlers (*n* = 53), boxers (*n* = 52) and fencers (*n* = 17). The fourth group (*strength* athletes) was composed of athletes (*n* = 182) with predominant anaerobic energy production (duration of exertion <1 minute, intensity of exertion submaximal to maximal): 100–400 m runners (*n* = 41), power lifters (*n* = 52), weightlifters (*n* = 44), throwers (*n* = 25) and jumpers (*n* = 20).

The Polish athlete population was stratified using the classification described by Druzhevskaya et al. [28]. The whole group of Polish athletes included 68 athletes classified as ‘top-elite’ (gold medallists in the World and European Championships, World Cups or Olympic Games) and 158 athletes classified as ‘elite’ (silver or bronze medallists in the World and European Championships, World Cups or Olympic Games). An additional 107 athletes were classified as ‘sub-elite’ (participants in international competitions). The others (*n* = 327) were classified as ‘non-elite’ athletes, all regional competitors with no less than 4 years of experience participating in their sports. Various methods were used to obtain the samples, including targeting national teams and providing information to national coaching staffs and athletes attending training camps.

Control samples were prepared from 684 unrelated sedentary volunteers (students of the University of Szczecin, aged 19–23, male *n* = 421 and female *n* = 263). All athletes and controls were Caucasian to reduce the possibility of racial gene skew and overcome any potential problems due to population stratification.

### Genetic analyses

The buccal cells donated by the subjects were collected in Resuspension Solution (GenElute Mammalian Genomic DNA Miniprep Kit, Sigma, Germany) with the use of sterile foam-tipped applicators (Puritan, USA). DNA was extracted from the buccal cells using a GenElute Mammalian Genomic DNA Miniprep Kit (Sigma, Germany) according to the manufacturer’s protocol. All samples were genotyped in duplicates using an allelic discrimination assay on a Rotor-Gene Real-Time Polymerase Chain Reaction (PCR) instrument (Corbett, Australia) with Taqman® probes. To discriminate *PPARG* Pro12 and 12Ala alleles (rs1801282), a TaqMan® Pre-Designed SNP Genotyping Assay was used (Applied Biosystems, USA) (assay ID: C___1129864_10), including primers and fluorescently labelled (FAM and VIC) MGB™ probes to detect both alleles. Genotypes were assigned using all of the data from the study simultaneously.

### Statistical analysis

The STATISTICA statistical package (version 8.0) was used to perform all statistical evaluations. Allele frequencies were determined by gene counting. A χ^2^ test was used to compare the *PPARG* Pro12Ala allele and genotype frequencies between the groups of athletes and control subjects. Bonferroni’s correction for multiple testing was applied, and the alpha level was determined by dividing the *P* value (0.05) by the number of comparisons performed (0.05/15). The level of statistical significance was set at *P*<0.003.

## Results

The results of the distribution of *PPARG* Pro12 and 12Ala variants in Polish male and female athletes (stratified into four groups according to the values of relative aerobic/anaerobic energy system contribution, time of competitive exercise performance and intensity of exertion in each sport) compared with male and female unfit controls are presented in Table 1. In both athletes and controls, the Pro12Ala genotype met Hardy-Weinberg expectations (*P* > 0.003 in all groups tested separately). The genotyping error was assessed as 1%, while the call rate (the proportion of samples in which the genotyping provided unambiguous reading) was above 95%.

**Table 1:** *PPARG* genotype distribution and frequencies of *PPARG* gene 12Ala allele in Polish athletes stratified by the values of relative aerobic/anaerobic energy system contribution, time of competitive exercise performance and intensity of exertion in each sport.

		Genotypes (%)					
Group	n	Pro12/Pro12	Pro12/12Ala	12Ala/12Ala	*P*	12Ala allele (%)	*P*
**ALL POLISH ATHLETES**	**660**	**535 (81.0)**	**112 (17.0)**	**13 (2.0)**	**0.035** a	**138 (10.4)**	**0.008** a
*Endurance athletes*	120	101 (84.2)	18 (15.0)	1 (0.8)	0.680 a	20 (8.3)	0.665 a
*Strength-endurance athletes*	162	135 (83.4)	25 (15.4)	2 (1.2)	0.592 a	29 (8.9)	0.391 a
*Sprint-strength athletes*	196	159 (81.1)	33 (16.8)	4 (2.1)	0.199 a	41 (10.4)	0.062 a
*Strength athletes*	182	140 (76.9)	36 (19.8)	6 (3.3)	0.006 a	48 (13.2)	0.0007 a *
**ALL POLISH CONTROLS**	**684**	**590 (86.3)**	**85 (12.4)**	**9 (1.3)**	**1.000**	**103 (7.5)**	**1.000**
**Male athletes**	**462**	**377 (81.6)**	**75 (16.2)**	**10 (2.2)**	**0.085** b	**95 (10.3)**	**0.019** b
*Endurance athletes*	99	83 (83.8)	15 (15.2)	1 (1.0)	0.671 b	17 (8.6)	0.480 b
*Strength-endurance athletes*	124	103 (83.1)	19 (15.3)	2 (1.6)	0.549 b	23 (9.3)	0.262 b
*Sprint-strength athletes*	137	110 (80.3)	24 (17.5)	3 (2.2)	0.154 b	30 (10.9)	0.044 b
*Strength athletes*	102	81 (79.4)	17 (16.7)	4 (3.9)	0.061 b	25 (12.2)	0.016 b
**Male controls**	**421**	**366**	**50**	**5**	**1.000**	**60 (7.1)**	**1.000**
**Female athletes**	**198**	**158 (79.8)**	**37 (18.7)**	**3 (1.5)**	**0.289** c	**43 (10.9)**	**0.165** c
*Endurance athletes*	21	18 (85.7)	3 (14.3)	0 (0.0)	0.846 c	3 (7.1)	0.813 c
*Strength-endurance athletes*	38	32 (84.2)	6 (15.8)	0 (0.0)	0.694 c	6 (7.9)	0.933 c
*Sprint-strength athletes*	59	49 (83.1)	9 (15.2)	1 (1.7)	0.919 c	11 (9.3)	0.685 c
*Strength athletes*	80	59 (73.8)	19 (23.7)	2 (2.5)	0.062 c	23 (14.4)	0.020 c
**Female controls**	**263**	**224**	**35**	**4**	**1.000**	**43 (8.2)**	**1.000**

a *P* values are calculated by χ^2^ test for comparisons between group of all athletes and control group as a whole.

b *P* values are calculated by χ^2^ test for comparisons between groups of male athletes and male controls.

c *P* values are calculated by χ^2^ test for comparisons between groups of female athletes and female controls.

A Bonferroni corrected alpha level was set at 0.003.

* Statistically significant differences (*P*<0.003).

**Figure 1:**
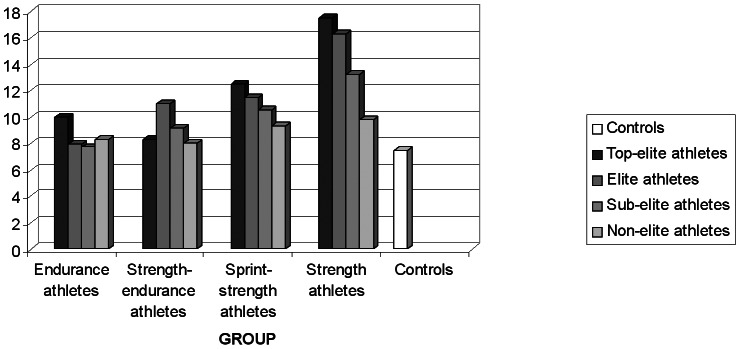
***PPARG*** **12Ala allele of the Pro12Ala polymorphism frequencies in four groups of Polish athletes stratified according to their level of competition and in controls.** Data are presented as relative values.

The genotype distribution of the Pro12Ala polymorphic site was not significantly different when all athletes (*P* = 0.035), or the distinct groups of athletes (*endurance*, *strength-endurance*, *sprint-strength* and *strength*) were compared to sedentary controls (*P* = 0.680, *P* = 0.592, *P* = 0.199, *P* = 0.006, respectively). There were no statistically significant differences in genotypes or allele frequencies between athletes and controls of different gender (Table 1). The data regarding *PPARG* 12Ala allele frequency in the four groups of Polish athletes stratified according to their level of competition are given in Figure 1. In the group of *strength* athletes stratified according to their level of competition the 12Ala allele frequencies were as follows: for top-elite athletes 17.5%, for elite athletes 16.3%, for elite athletes 13.2%, for non-elite athletes 9.8%. The *P* value for linear trend was *P* = 0.094.

The study revealed that the frequency of the minor 12Ala allele was higher in all Polish athletes than in controls; however, it failed to reach a statistically significant difference (10.4% vs. 7.5%; *P* = 0.008; Table 1). When considering the frequency of the 12Ala allele in the four groups of athletes, statistically significant differences were observed only in the *strength* athletes (13.2%; *P* = 0.0007). The statistically significant odds ratio of having the 12Ala allele (12Ala/12Ala + Pro12/12Ala genotypes vs. Pro12/Pro12 genotype; Table 2) for *strength* athletes was calculated as 1.88 (95% confidence interval 1.25–2.83; *P* = 0.002).

**Table 2:** The odds ratio (OD) of having the *PPARG* 12Ala allele (12Ala/12Ala + Pro12/12Ala genotypes vs. Pro12/Pro12 genotype) in Polish athletes stratified by the values of relative aerobic/anaerobic energy system contribution, time of competitive exercise performance and intensity of exertion in each sport.

Group	OD	95% confidence interval (CI)	*P*
**ALL POLISH ATHLETES**	1.47	1.10–1.96	0.010 a
*1. Endurance athletes*	1.18	0.69–2.02	0.544 a
*2. Strength-endurance athletes*	1.25	0.79–2.00	0.340 a
*3. Sprint-strength athletes*	1.46	0.96–2.22	0.075 a
*4. Strength athletes*	1.88	1.25–2.83	0.002 a *
**Male**	1.50	1.04–2.17	0.030 b
*1. Endurance athletes*	1.28	0.70–2.35	0.419 b
*2. Strength-endurance athletes*	1.36	0.78–2.35	0.274 b
*3. Sprint-strength athletes*	1.63	0.98–2.71	0.056 b
*4. Strength athletes*	1.73	0.99–3.01	0.053 b
**Female**	1.45	0.89–2.36	0.130 c
*1. Endurance athletes*	0.96	0.27–3.40	0.944 c
*2. Strength-endurance athletes*	1.08	0.42–2.75	0.877 c
*3. Sprint-strength athletes*	1.17	0.55–2.51	0.682 c
*4. Strength athletes*	2.04	1.12–3.74	0.019 c

a *P* values are calculated by χ^2^ test for comparisons between group of all athletes and control group as a whole.

b *P* values are calculated by χ^2^ test for comparisons between groups of male athletes and male controls.

c *P* values are calculated by χ^2^ test for comparisons between groups of female athletes and female controls.

A Bonferroni corrected alpha level was set at 0.003.

* Statistically significant differences (*P*<0.003).

## Discussion

The present study is the first report about the Pro12Ala polymorphism of the *PPARG* gene in Polish athletes. We examined the genotype distribution and allele frequency in four subgroups of athletes stratified by the values of relative aerobic/anaerobic energy system contribution, time of competitive exercise performance and intensity of exertion in each sport and the sedentary controls. We identified a significantly higher frequency of the *PPARG* 12Ala allele in the subgroup of the Polish athletes designated *strength* athletes compared to the frequency observed in the control participants. These results are in accordance with a previous study [26] showing that the 12Ala allele was more prevalent in the group of athletes (sprinters, throwers and weightlifters) that corresponds to our *strength* subgroup. Ahmetov et al. [26] also detected a hypertrophic effect of the *PPARG* 12Ala allele on muscle fibres, suggesting that the 12Ala allele is associated with the development and manifestation of the speed and force qualities. Moreover, the *PPARG* 12Ala allele was also overrepresented in a large cohort of Russian rowers [25], indicating the importance of the strength component in the overall performance of this strength-endurance discipline.

The functional relevance of the Pro12Ala amino acid change in the PPARγ2 protein is a results of its localisation within the PPARγ molecule. The C34G substitution in codon 12 of *PPARG* exon B causes the Pro12Ala polymorphism. It was first identified in 1997 [12] within the AF-1 domain of the amino terminus of the PPARγ2 protein that controls ligand-independent transcriptional activity [29]. The AF-1 domain contains a consensus MAPK (mitogen-activated protein kinase) site, and phosphorylation or sumoylation of this site reduces the PPARγ2 ligand-binding affinity [30], [31], [32]. Consequently, the potential of liganded PPARγ2 to activate the transcription of its target genes is decreased. Although position 12 in the PPARγ protein is not a consensus site for phosphorylation or sumoylation, the Pro12Ala change in AF-1 domain may indirectly facilitate the phosphorylation and/or sumoylation processes and thus be responsible for decreasing the PPARγ2 activity. To date, the detailed mechanism of such intramolecular interactions between the different amino acids of the AF-1 domain has remained unclear. Nevertheless, the association between the Pro12Ala polymorphism and the divergent transcriptional activity of PPARγ was confirmed during *in vitro* experiments. The estimation of the transcriptional activity of the 12Ala PPARγ2 variant, compared to the Pro12 variant, indicated that the *PPARG* 12Ala allele is associated with a less active form of PPARγ2 protein. Applied transient transfection assays revealed that the abilities of the 12Ala PPARγ2 protein to activate the transcription of prepared constructs containing PPRE [14] or specific genes, such as the *LPL* gene for lipoprotein lipase [13], were decreased. *In vitro* studies also indicated that the ability of the 12Ala PPARγ2 variant to induce adipogenesis in the presence of antidiabetic drugs was impaired [14]. These results were confirmed *in vivo* in association studies [33], [34], [35].

PPARγ2 is a transcriptional factor required for the proper expression of hundreds of genes engaged in cellular metabolism. The alterations in the activity of the PPARγ2 12Ala variant may be responsible for different physiological effects observed not only in adipocytes (where PPARγ2 is primarily expressed) but also in other tissues of the human body. The physiological needs of an athlete’s body require very subtle energy substrate regulation and mediation of the balance between fatty acid and glucose metabolism, especially in terms of metabolic stress for prolonged exertion or short-term, very intense exercises. PPARγ2 acts as a molecular sensor that controls the metabolism and transport of fatty acids in different tissues; it may thus influence the energy substrate selection. Biochemical studies revealed that, under physiological conditions, PPARγ agonists promote a flux of fatty acids into adipose tissue and away from skeletal muscle and liver. These processes result in a decrease of fatty acid metabolism in the aforementioned tissues and a consequent increase of glucose utilisation via the Randle cycle in muscles and a reduction of glucose production in the liver [36]. Most of these effects are mediated in a complicated network via insulin-dependent signalling pathways.

One of the first reports regarding the physiological relevance of the *PPARG* Pro12Ala polymorphism was the study by Deeb, et al. [13] that indicated that the insulin sensitivity was significantly greater in non-diabetic 12Ala allele carriers. The positive association between the *PPARG* 12Ala allele and improved insulin sensitivity was confirmed in many studies performed worldwide in obese and non-obese individuals [19], [37]. However, the insulin-sensitising effect of the *PPARG* 12Ala allele on skeletal muscle is either lost or masked by insulin resistance in patients with type 2 diabetes mellitus [21]. The increase in insulin sensitivity was coupled to the intensity of lipolysis: in adipose tissue, the influence of enhanced insulin on the suppression of lipolysis results in a decreased release of FFAs (Free Fatty Acids) [38]. However, it remains unclear which specific genes relevant for insulin-dependent FFA release suppression are definitely affected by the transcriptional changes resulting from *PPARG* Pro12Ala polymorphism. The group of proposed candidates includes the genes for adipose tissue-derived adipokines (e.g., tumour necrosis factor-α (TNF-α), resistin, leptin and adiponectin) that are under the transcriptional control of PPARγ and have been associated with insulin sensitivity [4].

The secondary effect of *PPARG* 12Ala-mediated improvement of insulin sensitivity may be observed in muscle cells. At first glance, this may seem surprising because PPARγ2 is minimally expressed in the skeletal muscles, but there are some physiological explanations for this fact. The insulin-induced inhibition of lipolysis in adipocytes results in reduced plasma FFA availability, which may favour using glucose in muscle cells. This specific shift of energy balance towards glucose utilisation rather than FFA mobilisation upon insulin stimulation [38] seems to be more efficient in *PPARG* 12Ala carriers due to the improved insulin sensitivity observed in such individuals. This assumption was confirmed in a study in which the effect of decreasing the lipid oxidation with an accompanying increase of cellular glucose metabolism after insulin stimulation was mainly observed in lean subjects carrying the 12Ala allele, while the Pro12Pro12 homozygotes revealed significantly lower substrate flexibility [39]. The direct non-invasive measurements of blood flow and glucose uptake in human skeletal muscles and adipose tissue also revealed that the rates of muscle glucose uptake were significantly higher in nonobese 12Ala allele carriers than in Pro12Pro12 individuals [20].

Increased glucose utilisation in working skeletal muscles may be one of the key elements crucial for athletes performing short-term exercises, such as the representatives of the *strength* subgroup analysed in the presented study. For athletes who perform sports that involve lifting, jumping, throwing, and short sprints, the anaerobic system is regarded as a fundamental mechanism of energy production. In anaerobic metabolism, glucose is the most important fuel, as it is needed for glycolysis to provide the amount of energy required for very short (approximately 20–30 s) and very intense exertion.

The aforementioned flexibility of energy substrate usage is an element that is unquestionably crucial for performing the physical exertions characteristic of athletes. However, body mass and composition can be considered equally important factors in athletic performance. Because PPARγ regulates adipocyte differentiation and controls body fat storage, the relevance of the PPARγ polymorphism in the context of susceptibility to obesity is of major interest. The different consequences of carrying the *PPARG* 12Ala allele on BMI were observed in overweight/obese and lean subjects [40], [41]. A meta-analysis of 40 datasets from 30 independent studies revealed that the *PPARG* Pro12Ala polymorphism had an effect on BMI in individuals with marked obesity (12Ala carriers had a higher BMI than Pro12 homozygotes), while this effect was not observed in lean subjects [19]. These findings indicate that the Pro12Ala polymorphism modulates body weight, but its impact is modified by other genetic components (e.g., the Trp64Arg polymorphism in the *ADRB3* gene; [42]) and environmental factors, such as different dietary habits or physical activity levels. The low dietary intake of polyunsaturated fatty acids favoured BMI increases in 12Ala allele carriers more than in Pro12Pro12 homozygotes; when the ratio of polyunsaturated to saturated fatty acids was high, the reverse effect on BMI was observed [43]. The effect of dietary fatty acid intake on BMI may be modified by physical activity. A study on non-diabetic subjects indicated that the beneficial additive effects of physical exercise and a healthy (i.e., rich in polyunsaturated fatty acids) diet are restricted to *PPARG* Pro12Pro12 homozygotes. In 12Ala allele carriers, the relationships between diet, activity level and body weight are more complicated: the beneficial effects are only observed when the polyunsaturated to saturated fatty acid ratio and physical activity are simultaneously elevated. In other cases (e.g., subjects on a healthy diet, inactive or active subjects exposed to a high-fat diet), the 12Ala carriers displayed effects that were comparable to those observed in Pro12Pro12 individuals who were completely inactive and consumed a diet rich in saturated fatty acids [44].

These data may suggest that the *PPARG* 12Ala allele is positively associated with a susceptibility to obesity; however, the observed effects of its presence in an individual’s genotype strongly depend on that individual’s lifestyle behaviours. Taking these findings into consideration, one main conclusion for athletes seems to be particularly important: to develop a favourable weight-to-strength ratio in professional athletes who are *PPARG* 12Ala allele carriers, strict dietary discipline should be maintained. This is likely to be especially important for athletes competing in sports that involve lifting, jumping, throwing and short sprints, for whom strength abilities are essential. In our study, the *strength* subgroup of analysed athletes is characterised by a higher frequency of the *PPARG* 12Ala allele compared to sedentary controls. All athletic participants from this group undergo a dietary regime. For physically active 12Ala allele carriers, a strict diet seems to be the crucial environmental factor that favourably modulates the influence of their genetic components, which most likely enables them to achieve a high performance level. We tend to believe that the proper combination of genotype, training and diet is most likely responsible for developing the appropriate relations between body mass and strength in the athletes from the *strength* subgroup analysed in the presented study.

The role of PPARγ in athletic performance is multifarious because PPARγ also regulates bone mass, which is a phenotype trait that creates a structural scaffold crucial for effective load transfer in athletes. There is evidence for an antiosteogenic action of PPARγ. Natural and synthetic PPARγ ligands were recognised as factors inhibiting osteoblast differentiation and bone formation in mice [45]. The study of PPARγ-deficient mice as well as *in vitro* experiments revealed that PPARγ haploinsufficiency promotes osteoblastogenesis [46] and enhances bone development. The osteogenic effect observed in the absence of PPARγ is most likely connected with a switch of the mesenchymal precursor cell differentiation program towards osteoblasts rather than adipocytes. The reduced transcriptional activity of PPARγ results in the decreased expression of PPARγ target genes coding for antiosteogenic-signalling factors [47]. Based on data obtained in mouse models, the reduction of PPARγ activity associated with the Pro12Ala polymorphism could enhance osteoblastogenesis, resulting in increased bone mass in humans. Thus, athletes carrying the *PPARG* 12Ala allele might benefit from having stronger bones that are better adjusted to withstand extreme forces and transfer loads that are over the normal loading conditions. This aspect is especially important for athletes performing strength sports such as powerlifting or weightlifting, for which tremendous weight loads are transferred throughout the whole training program and during competition.

The diverse data obtained for the Pro12Ala polymorphism in different human populations indicate the possibility that this variation within the *PPARG* gene does not influence physiological traits alone, implying that multiple gene-environment interactions may contribute to the observed differential effects of the 12Ala allele. There may be other functional polymorphisms in *PPARG* regulatory regions that reduce PPARγ transcriptional activity and are in linkage disequilibrium with the *PPARG* Pro12Ala gene variant [48].

Our study has limitations. We are aware that the number of participants in the analysed groups of athletes is relatively small; however, because there are a limited number of elite athletes worldwide and in Poland, we were unable to gather additional samples for this population. An excess of the *PPARG* 12Ala allele was observed in the *strength* athlete subgroup, indicating a favourable influence of the 12Ala allele with respect to strength athlete status; however, linking only one SNP (e.g., C34G in the *PPARG* gene causing the Pro12Ala polymorphism) to athletic performance could be largely questioned. Therefore, these results should be interpreted with caution, and the analysis of the association between the *PPARG* Pro12Ala polymorphism and athletic performance needs to be replicated in additional large-scale prospective studies.

In conclusion, the results of our study indicate a statistically significant higher frequency of the *PPARG* 12Ala allele (compared to sedentary controls) in the subgroup of *strength* athletes performing short-term and very intense exertion characterised by predominant anaerobic energy production. Taking into account the results of a previous report [26], showing muscle hypertrophic effects in non-athletic 12Ala allele carriers, and the increased frequency of this variant in a group of Russian speed and force athletes as well as studies confirming different transcriptional activities of the Pro12 and 12Ala PPARγ2 protein variants [13], [14] causing divergent physiological effects, the *PPARG* 12Ala allele may be a relevant genetic factor favouring strength abilities in professional athletes, especially in terms of insulin-dependent metabolism, a shift of the energy balance towards glucose utilisation and the development of a favourable weight-to-strength ratio. The most recent findings [49], [50] show that the group of genetic components that are believed to play a role in strength athletic abilities is still growing, indicating that genetic backgrounds play an important role in developing the muscular strength. The analysis of *PPARG* polymorphism along with other gene variations may give an opportunity to use it in sports selection.
